# Serological and Molecular Evaluation of *Leishmania infantum* Infection in Stray Cats in a Nonendemic Area in Northern Italy

**DOI:** 10.5402/2013/916376

**Published:** 2013-07-07

**Authors:** Eva Spada, Daniela Proverbio, Antonella Migliazzo, Alessandra Della Pepa, Roberta Perego, Giada Bagnagatti De Giorgi

**Affiliations:** ^1^Dipartimento di Scienze Veterinarie per la Salute, la Produzione Animale e la Sicurezza Alimentare, Università degli Studi di Milano, Via G. Celoria 10, 20133 Milano, Italy; ^2^Centro di Referenza Nazionale per le Leishmaniosi, Istituto Zooprofilattico Sperimentale della Sicilia, Via R. Dicillo 4, 90129 Palermo, Italy

## Abstract

Infection by *Leishmania* species is increasing worldwide. It was hypothesized recently that cats act as a secondary reservoir for *Leishmania* infection. The aim of the present study was to assess the prevalence of *Leishmania infantum* antibodies and DNA in blood samples collected in a sample of stray cats in metropolitan area of Milan in northern Italy, which is a nonendemic area for leishmaniasis. An indirect immunofluorescence antibody test for *L. infantum* showed that 59 of 233 cats (25.3%) were seroreactive, 38 samples (16.3%) had antibody titers of 1 : 40, 15 (6.4%) had antibody titers of 1 : 80, and 6 (2.6%) had antibody titers of 1 : 160. Feline immunodeficiency virus (FIV) seropositive status was statistically associated with seroreactivity to *L. infantum* (*P* = 0.01) as shown by univariate and multivariate logistic regression (*P* = 0.0098; OR = 7.34). All blood samples that were tested using real-time PCR were negative for parasite DNA. These results were surprising, since no autochthonous human or canine cases of leishmaniasis have ever been reported in this region of northern Italy. It is possible that this high seroreactivity to *L. infantum* could be due to cross-reaction with antigens from other parasites. Additional studies that include parasite isolation are needed to clarify our findings on feline leishmaniasis in this region.

## 1. Introduction

Leishmaniasis in the Old World is caused by the protozoa *Leishmania infantum*. It is prevalent in countries in the Mediterranean basin, and dogs are the main reservoir of the parasite in that region [1]. In recent years, autochthonous cases of human and canine disease have been recorded at higher latitudes, namely, in Germany [2, 3], The Netherlands [4], and North America [5]. Infections have also been reported in species other than dogs and humans, including horses [6] and cows [7]. There have been numerous reports of feline leishmaniasis (FeL), mostly in cats living in known endemic areas [8–10]; some of the cats had concurrent immunosuppressive infections [9–11]. In countries in southern Europe, where canine leishmaniasis (CanL) is endemic, serological investigations of feline populations have revealed seroprevalence rates ranging from less than 1% to more than 60% [9–21]. Given the diffusion of *Leishmania *infection and the lack of information regarding infection rates in cats in the Milan metropolitan area in northern Italy, the aim of the present study was to assess the prevalence of leishmaniasis in a large representative sample of stray cats from this nonendemic area. A secondary aim was to analyze the results according to clinical, laboratory and infectious data.

## 2. Materials and Methods

### 2.1. Feline Population

During a 2-year period (January 2008 to January 2010), blood samples were collected from 233 European shorthair stray cats from urban colonies in Milan, northern Italy, during a trap-neuter-release (TNR) program that was approved by the local authority of the city council. The program was conducted as described previously [22]. 

### 2.2. Data Collection

The following data were recorded: sex (*n* = 233), age (*n* = 233), body condition score (BCS) (*n* = 215), area of colony of provenance, that is, one of the seven municipalities of Milan (*n* = 233), health status based on physical examination (*n* = 233), and dermatological evaluation (*n* = 121). Cats were classified as healthy or unhealthy depending on the clinical findings (Table 1).

### 2.3. Sample Collection

Whole blood samples were collected by cephalic or jugular venipuncture into tubes with EDTA anticoagulant for complete blood cell (CBC) count and polymerase chain reaction (PCR) testing and into empty tubes for serology. All samples used for serology and PCR were stored at −20°C until use. 

### 2.4. Hematological and Serological Examination

Within 24 hours of sample collection, a CBC count was performed on whole blood (*n* = 127) using an ADVIA 120 System (Siemens Healthcare Diagnostics, Milan, Italy). Cats were categorized as having alterations in the CBC as shown in Table 1. 

Serological assessment was performed to determine the presence of the following: antibodies to the feline immunodeficiency virus (FIV) relative to the gp40 and p24 FIV antigens, the feline leukemia virus (FeLV) p27 antigen (Snap FeLV/FIV Combo Plus Test, Idexx Laboratories, Hoofddorp, The Netherlands) (*n* = 137), and *Toxoplasma gondii* IgG antibodies (IFAT, Fuller Laboratories, Fullerton, CA, USA) (*n* = 79). The results of these serological tests have been already published [22] and were reanalyzed with the present results. 

For various technical reasons, not all data were available for all 233 cats.

### 2.5. Indirect Immunofluorescence Antibody Test

The presence of anti-*L. infantum* antibodies was measured by an indirect immunofluorescence antibody test (IFAT) performed according to the recommendations of OIE [23] using MHOM/IT/80/IPT1 as a whole-parasite antigen fixed on multispot slides (Bio Merieux Spa, Florence, Italy) and fluorescently-labeled antifeline gamma globulin (Sigma Aldrich, Milan, Italy) as conjugate. Positive sera were diluted serially and tested to establish the maximum reaction titer, starting at a dilution of 1 : 40. Positive and negative controls were included on each slide. 

### 2.6. PCR

*L. infantum* DNA was amplified from 200 *μ*L of whole blood by real-time PCR using the Illustra Blood genomicPrep Mini Spin kit (GE Healthcare, Milan, Italy) following the manufacturer's instructions. The target for amplification was a 116-bp fragment in the constant region of the kDNA minicircle of *L. infantum*. This is one of the kDNA minicircle families that is used to identify the *Leishmania* genus. The primers used were QLK2-UP 5′-GGCGTTCTGCGAAAACCG-3′  and  QLK2-DOWN  5′-AAAATGGCATTTTCGGGCC-3′;  the TaqManprobes were Q Leish Probe 2 and 5′-FAM TGGGTGCAGAAATCCCGTTCA-3′- Black Hole. 

### 2.7. Statistical Analysis

Univariate analysis of the categorical data was performed using the chi-square test or Fisher's exact test. Any parameters statistically linked to IFAT seroreactivity for *L. infantum *or to the presence of *L. infantum *DNA as detected by PCR were used in a logistic regression model to test for independent risk factors associated with the *L. infantum* positivity. Associations were considered statistically significant when *P* < 0.05; both the *P* value and odds ratio (OR) are reported. Data were analyzed using MedCalc Software (version 12.3.0; Mariakerke, Belgium).

## 3. Results 

**Figure 1:**
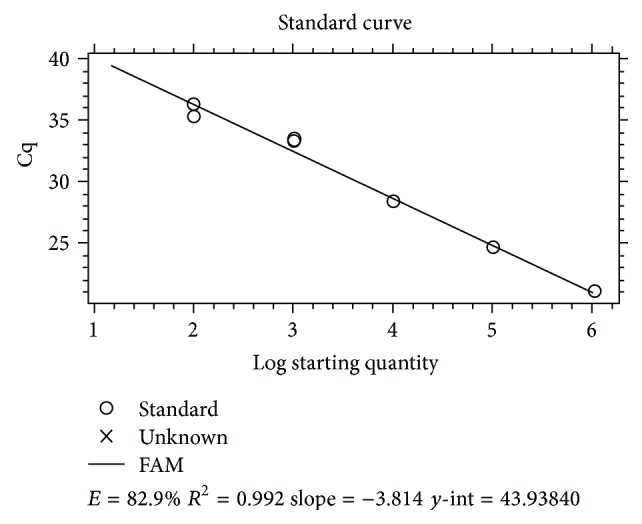
Standard curve in logarithmic scale.

**Figure 2:**
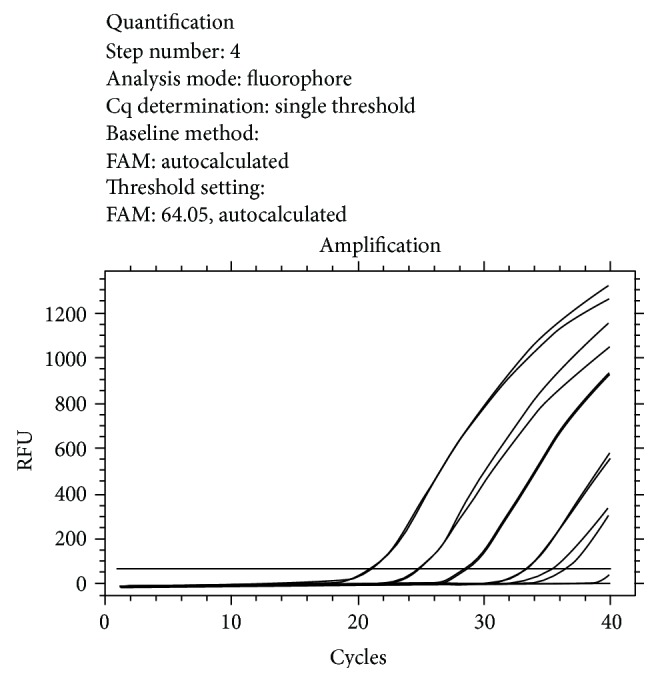
Amplification curve: amplification of the standards (from 10^6^ Leish/mL to 100 Leish/mL). Below the threshold the nonamplified samples (negative).

The characteristics of the feline study population are summarized in Table 1. The serology test for *L. infantum *showed that 25.3% (59/233) of the cats had *L. infantum* seroreactivity, 38 (16.3%) had antibody titers of 1 : 40, 15 (6.4%) had titers of 1 : 80, and 6 (2.6%) had antibody titers of 1 : 160. All blood samples tested using real-time PCR were negative for the presence of *L. infantum* DNA. Standard curve and amplification curve of real-time PCR were reported in Figures 1 and 2, respectively. 

No statistical association was found between seroreactivity to *L. infantum* and age, sex, BCS, municipality of provenance, clinical finding, dermatological findings or FeLV, and *T. gondii* serology. In contrast, in terms of CBC, neutrophilia was statistically associated with seroreactivity to *L. infantum* (*P* = 0.01) in univariate analysis, but this association was not confirmed using multivariate logistic regression (*P* = 0.57). In terms of serology for the retrovirus, FIV seropositive status was statistically associated with seroreactivity to *L. infantum* (*P* = 0.01). This association was confirmed by multivariate logistic regression: *P* = 0.0098 and OR = 7.34 (95%CI = 1.96 to 27.59). The distribution of the parameters that were evaluated and compared in *L. infantum* seropositive and seronegative cats is shown in Table 1. 

## 4. Discussion 

This study is the first epidemiological investigation of feline *Leishmania* infection in the metropolitan area of Milan, which is a nonendemic area for leishmaniasis. We found seroreactivity to *L. infantum* by IFAT in 59 of the 233 (25.3%) stray cats that we examined. These results were surprising, since no autochthonous human or canine cases of leishmaniasis have ever been reported in this region in northern Italy. In countries in southern Europe where leishmaniasis is endemic, serological investigations performed in feline populations using different techniques have revealed prevalence rates that range from less than 1% to more than 60% [9–21]. In particular, the seroprevalence in Italy ranges from 0.9% to 68% [9–11, 13], in Spain from 3.7% to 60% [14–16], and in Portugal from 0.6% to 2.8% [18–20]. In Greece, the seroprevalence is 3.9% [17] and in France it is 12.4% [12]. These results in *L. infantum* endemic geographical regions may reflect differences in the serological techniques used, in the cut-off values or positive thresholds and in the populations of cats that were tested. As here, previous epidemiological studies have used IFAT to detect antibodies to *Leishmania *spp. in cats. An important concern is that there is no standardized IFAT method for serological evaluation of antibodies to *Leishmania* spp. in cats; accordingly, there is no universally accepted antibody titer cut-off value that corresponds to active infection. Cut-off titers validated in dogs are used frequently for cats, but the immune response could be different in cats than in dogs. 

None of the peripheral blood samples we examined using real-time PCR were positive for parasite DNA. PCR has been used previously by others, either alone or in combination with serology, as in our study, to assess the prevalence of feline *Leishmania* infection [9, 11, 14, 24, 25]. Blood is not the best specimen for PCR diagnosis of leishmaniasis. Specifically, PCR performed on canine blood has lower sensitivity, specificity, and positive and negative predictive values compared to PCR performed on canine lymph node aspirates [26], and this may be true for samples from cats as well. However, blood sampling is less invasive and is easy to perform, particularly for epidemiological studies involving numerous subjects, as in our survey. 

Although dogs have been universally regarded as the domestic reservoir hosts of zoonotic visceral leishmaniasis caused by *L. infantum*, some researchers have hypothesized that cats may also act as a secondary reservoir host of leishmaniasis rather than simply as an accidental host [9, 14, 15]. Differences in immune response, vector host preference, or innate resistance in cats to vector-borne diseases could account for the observed differences in the prevalence of infection in canine versus feline populations in endemic areas. Immunosuppressive agents, such as FIV or FeLV, or disease and stress, can induce immunological dysfunction and impair the cellular immune response. This allows active multiplication of the parasite and widespread visceral dissemination of the protozoa [27]. In our survey, FIV infection was statistically associated with seroreactivity to *L. infantum* by IFAT, and FIV-positive cats were 7.3 times more likely to be *L. infantum* seroreactive than FIV-negative cats (*P* = 0.0098). This association has also been found in previous studies performed in endemic area of Southern Italy [9, 11]. 

Based on results from a recent survey, continental northern Italy is now focally endemic for leishmaniasis, but no sand-flies (vector) or autochthonous cases of human and canine leishmaniasis have been identified in Milan or its suburbs [28]. Cases of CanL are commonly diagnosed in the area where we performed our study, but the histories of the affected dogs always reveal that they have lived or travelled in areas that are endemic for CanL [29, 30]. A canine epidemiological survey of 313 dogs in a public animal shelter that were tested for *L. infantum* by IFAT more than 10 years ago (2002-2003) in the urban area of Milan found a seroprevalence of 3.4% [31] Although the history of dogs in animal shelters is often unknown, some of these dogs may have come from areas that are endemic for *L. infantum *infection. In contrast, it is unlikely that all of the *Leishmania* seropositive cats found in our study population were infected in endemic areas. In the present study, the feline seroprevalence for *L. infantum* was much higher than the canine seroprevalence found 10 years previously in a canine population in an animal shelter in the same area. Notably, this area is still considered nonendemic for leishmaniasis. We speculate that the serology results for leishmaniasis in our survey may be an overestimation due to the possibility of IFAT cross-reactivity between *L. infantum* and other pathogens. Cross-reactivity with other pathogens is possible on some serologic tests, especially those that use a whole-parasite antigen, as we did here. There was no significant correlation between* T. gondii* positivity and *L. infantum* positivity in our study. This may suggest a lack of cross-reactivity with *Toxoplasma* parasites. New vector-borne parasites have been found that affect cats, such as *Ehrlichia *spp., *Rickettsia felis*, *Anaplasma phagocytophilum*, and *Babesia* spp. (according to the vector-borne disease ESCCAP guidelines) [32] that might be able to cross-react with *Leishmania*. This has been demonstrated in dogs in that IFAT cross-reactivity has been reported for *L. infantum* and *Trypanosoma cruzi*, *Leishmania braziliensis*, and *Ehrlichia canis* infection [33].

## 5. Conclusions

Our results demonstrate high levels of seroreactivity to *L. infantum* in cats in an area of northern Italy that has traditionally been considered to be free of leishmaniasis and nonendemic for this infection in dogs. Possible IFAT cross-reactivity and a lack of a validated serological method for feline specie could explain our unexpectedly high seroprevalence. Additional studies that include parasite isolation are needed to clarify our findings on feline leishmaniasis in this geographic area. 

## Figures and Tables

**Table 1:** Characteristics of a population of stray cats in northern Italy and a comparison of characteristics in *Leishmania infantum* seropositive versus seronegative cats as determined using an indirect immunofluorescence antibody test.

Factor	Category	Total population	Sero positive	Sero negative	Univariate *P*-value
Age	Young (≤6 months)	106 (45.5%)	24 (40.7%)	82 (47.1%)	0.4788
	Adult (>6 months)	127 (54.5%)	35 (59.3%)	92 (52.9%)	

Sex	Female	153 (65.7%)	38 (64.4%)	115 (66.1%)	0.9387
	Male	80 (34.3%)	21 (35.6%)	59 (33.9%)	

BCS	Scarce (≤3/9)	19 (8.8%)	4 (7.4%)	15 (9.3%)	0.8802
	Good (≥4/9)	196 (91.2%)	50 (92.6%)	146 (90.7%)	

Colony of origin	Zone 2	11 (4.7%)	2 (3.4%)	9 (5.2%)	0.0825
	Zone 4	95 (40.8%)	18 (30.5%)	77 (44.2%)	
	Zone 5	9 (3.9%)	0 (0.0%)	9 (5.2%)	
	Zone 6	23 (9.9%)	8 (13.6%)	15 (8.6%)	
	Zone 7	53 (22.7%)	17 (28.8%)	36 (20.7%)	
	Zone 8	21 (9.0%)	5 (8.5%)	16 (9.2%)	
	Zone 9	21 (9.0%)	9 (15.2%)	12 (6.9%)	

Clinical examination	Healthy	49 (21.0%)	12 (20.3%)	37 (21.3%)	0.9728
	Unhealthy	184 (79.0%)	47 (79.7%)	137 (78.7%)	
	Stomatitis	92 (39.5%)	17 (28.8%)	75 (43.1%)	0.0740
	Ocular discharge	35 (15.0%)	10 (16.9%)	25 (14.4%)	0.7881
	Nasal discharge	21 (9.0%)	5 (8.5%)	16 (9.2%)	0.9236
	Pale mucous membranes	12 (5.2%)	4 (6.8%)	8 (4.6%)	0.7532
	Lymphadenomegaly	117 (50.2%)	30 (50.8%)	87 (50%)	0.9696

Dermatological examination	Absence of lesions	83 (68.6%)	17 (54.8%)	66 (73.3%)	0.0912
	Presence of lesions	38 (31.4%)	14 (45.2%)	24 (26.7%)	
	Crusted dermatitis	22 (18.2%)	7 (22.6%)	15 (16.7%)	0.6410
	Scaling	5 (4.1%)	1 (3.2%)	4 (4.4%)	0.8188
	Nodular dermatitis	3 (2.5%)	2 (6.5%)	1 (1.1%)	0.3273
	Alopecia	18 (14.9%)	8 (25.8%)	10 (11.1%)	0.0910
	Ectoparasites	27 (22.3%)	6 (22.2%)	21 (77.8%)	0.8346
	Dermatophytosis	9 (7.4%)	1 (3.2%)	8 (8.9%)	0.5225

CBC results	Absence of anemia	29 (22.8%)	5 (16.7%)	24 (24.7%)	0.5015
	Presence of anemia	98 (77.2%)	25 (83.3%)	73 (75.3%)	
	Decreased Ht	97 (76.4%)	25 (83.3%)	72 (74.2%)	0.4352
	Decreased Hb	23 (18.1%)	7 (23.3%)	16 (16.5%)	0.5627
	Decreased RBC	41 (32.3%)	11 (36.7%)	30 (30.9%)	0.7158
	Thrombocytopenia	10 (7.9%)	2 (7.7%)	8 (8.2%)	0.9149
	Leukocytosis	5 (3.9%)	2 (6.7%)	3 (3.1%)	0.7319
	Leukopenia	15 (11.8%)	3 (10.0%)	12 (12.4%)	0.9776
	Neutrophilia	15 (11.8%)	8 (26.7%)	7 (7.2%)	**0.01** (0.57)∗
	Neutropenia	2 (1.6%)	0 (0.0%)	2 (2.1%)	0.9631
	Lymphocytosis	2 (1.6%)	0 (0.0%)	2 (2.1%)	0.9631
	Lymphopenia	33 (26.0%)	12 (40.0%)	21 (21.6%)	0.0776
	Eosinophilia	12 (9.4%)	4 (13.3%)	8 (8.2%)	0.6346
	Eosinopenia	33 (26.0%)	9 (30.0%)	24 (24.7%)	0.7371

FIV status	Positive	12 (8.8%)	7 (21.2%)	5 (4.8%)	**0.01 (0.0098)** ^*^
	Negative	125 (91.2%)	26 (78.8%)	99 (95.2%)	OR = 7.34∗ (95% CI 1.96–27.59)

FeLV status	Positive	5 (3.6%)	0 (0.0%)	5 (4.8%)	0.453
	Negative	132 (96.4%)	33 (100.0%)	99 (95.2%)	

*T. gondii* status	Positive	26 (32.9%)	9 (36.0%)	17 (31.5%)	0.8886
	Negative	53 (67.1%)	16 (64.0%)	37 (68.5)	

BCS: body condition score; CBC: complete blood count; Ht: hematocrit, Hb: hemoglobin, RBC: red blood cells, FIV: Feline immunodeficiency virus; FeLV: Feline Leukemia virus; OR: odds ratio; CI: confidence interval.

*P*-values in bold are statistically significant (*P* < 0.05).

^*^Results from multivariate logistic regression analysis.
